# Translating Science Into Practice: The Perspective of the Doha 2019 IAAF World Championships in the Heat

**DOI:** 10.3389/fspor.2019.00039

**Published:** 2019-09-27

**Authors:** Sebastien Racinais, Douglas Casa, Franck Brocherie, Mohammed Ihsan

**Affiliations:** ^1^Aspetar Orthopaedic and Sports Medicine Hospital, Doha, Qatar; ^2^Department of Kinesiology, Korey Stringer Institute, University of Connecticut, Storrs, CT, United States; ^3^Laboratory Sport, Expertise and Performance, French Institute of Sport (INSEP), Paris, France

**Keywords:** heat acclimatization, hydration, acclimation, pre-cooling, exertional heat illness

## Abstract

Hot and humid ambient conditions may play a major role during the endurance events of the 2019 IAAF world championships, the 2020 summer Olympics and many other sports events. Here, various countermeasures with scientific evidence are put in perspective of their practical application. This manuscript is not a comprehensive review, but rather a set of applied recommendations built upon sound scientific reasoning and experience with elite athletes. The primary recommendation for an athlete who will be competing in the heat, will be to train in the heat. This acclimatization phase should last for 2 weeks and be programmed to accommodate the taper and travel requirements. Despite extensive laboratory-based research, hydration strategies within athletics are generally dictated by the race characteristics. The main opportunities for hydration are during the preparation and recovery phases. In competition, depending on thirst, feeling, and energy requirements, water may be ingested or poured. The athletes should also adapt their warm-up routines to the environmental conditions, as it may do more harm than good. Avoiding harm includes limiting unnecessary heat exposure before the event, warming-up with cooling aids such as ice-vest or cold/iced drinks, and avoiding clothing or accessories limiting sweat evaporation. From a medical perspective, exertional heat stroke should be considered immediately when an athlete collapses or struggles during exercise in the heat with central nervous system disorders. Once a rectal temperature >40.5°C is confirmed, cooling (via cold water immersion) should be undertaken as soon as possible (cool first/transport second).

## Introduction

The 17th IAAF World Championships will take place in Doha, from the 27th September to the 6th October 2019. While the track and field events will be held in an AC stadium, the road races will be held on the seaside, at night time. A retrospective analysis of 36-years of MERRA-2 meteorological data over the competition period indicates that weather conditions are typically both hot and humid during this time of the year, with average maximum daytime temperatures reaching 38°C, and average daily dewpoint temperatures approaching 22°C. The temperature peaks early afternoon (range 33–42°C) and is the lowest at night (range 23–31°C). The WBGT follows a similar pattern with a mid-day peak (average 33°C, range 27–44°C) and an overnight minimum (average 24°C, range 19–28°C). Such conditions impair endurance performance and increase the risk of exertional heat illness (Racinais et al., 2015a). As such, understanding the mitigating factors surrounding human thermoregulation, knowledge, and practice of effective countermeasure strategies and medical care are imperative to ensure adequate and safe athlete preparation and performance (Alhadad et al., 2019). This manuscript briefly puts in perspective the main countermeasures to mitigate heat stress considering their scientific evidence as well as their practicability during the athletic championship.

## Training in the Heat: Failing to Plan is Planning to Fail

**Figure 1:**
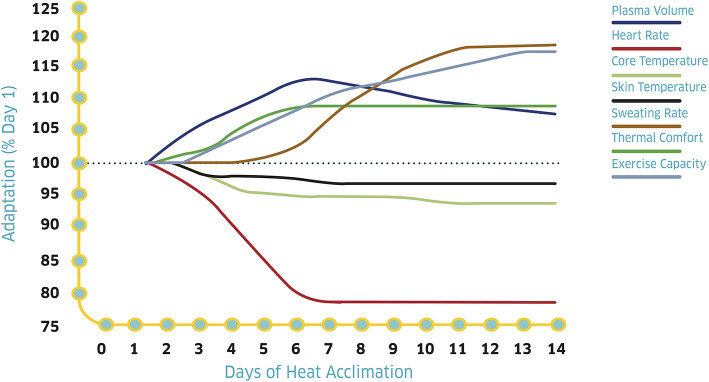
Time course of heat acclimation adaptations with repeated training in the heat. Adapted with permission from Périard et al. (2015).

Heat stress has been shown to dramatically decrease endurance performance, but this decrement can be progressively attenuated with the acclimatization induced by repeated training in the heat (Racinais et al., 2015b). Importantly, heat acclimatization may also reduce the likelihood of suffering from heat illnesses. As such, heat acclimatization should be a priority before any event where the ambient conditions may be hot and/or humid, even if the level of heat stress may be mild or uncertain. Indeed, heat acclimatization does not impair performance in cooler environments and may even improve it under some circumstances (Lorenzo et al., 2010; Racinais et al., 2014). Briefly, the most visible adaptations of the body to repeated training in the heat include an increased sweat rate, a decreased heart rate at a given intensity, a better retention of electrolytes, and a decreased body core temperature (Figure 1; Périard et al., 2015; Tyler et al., 2016; Daanen et al., 2018).

**Figure 2:**
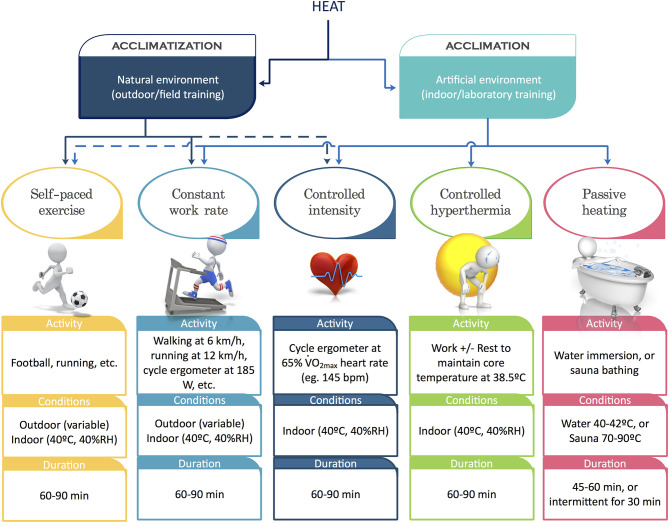
Various methods for heat acclimatization/acclimation. Adapted with permission from www.ephysiol.com. RH, relative humidity; W, watt; bpm, beat per minutes.

Intuitively, it can be reasoned that the most specific adaptations would be conferred by training in the same environment as the upcoming competition. However, where such an undertaking may not be possible, the use of artificial heat exposure such as training in a hot room, sauna bathing, hot bath post thermoneutral training are potentially some useful alternatives (Figure 2). The fundamental requisite for an effective heat acclimation involves substantial and repeated increases in core temperature, skin temperature, and sweating. In addition, the relative success of a heat adaptation program depends on the specificity (active vs. passive) and frequency of the exposure. It is ideal that at least 60–90 min of exercise is undertaken in the heat to confer rapid adaptations (Tyler et al., 2016), and a total period of 2 weeks is allocated to facilitate maximal adaptations (Racinais et al., 2015b). Additional training sessions may be done in cooler environments, whilst it is recommended that sleep and recovery should always be in a cool environment. When training in the heat, the relative intensity can be controlled by heart rate throughout acclimation (Périard et al., 2015). A decrease in absolute training intensity is usually evident during the initial days of heat acclimation, and it is possible to commence the acclimation program at reduced thermal load (i.e., lower environmental temperatures, exercise duration or intensity). If training in the heat includes high-intensity workouts with a neuromuscular focus, this should be done at the beginning of the session, before athletes attain elevated body temperatures (Karlsen et al., 2015b).

The time required to achieve optimal acclimatization may vary but most adaptations have been shown to develop within 7–10 days, with 14 days being preferable (Karlsen et al., 2015a; Périard et al., 2015; Racinais et al., 2015b; Tyler et al., 2016). It is thus generally recommended that 2 weeks of heat acclimation be undertaken prior to competing in hot and/or humid ambient conditions (Racinais et al., 2015a). Adaptations gathered following heat acclimation have been shown to decay in a symmetrical fashion, but with probably a slower pace. Indeed, most adaptations have been shown to decline following 1–2 weeks, but some benefits can be maintained for up to 1 month (Daanen et al., 2018). The rate of decay can be prolonged by including heat exposures post-acclimation/acclimatization. Importantly, re-acclimation following a dedicated period of heat-acclimation has been shown to be more accelerated compared with the initial rate of acclimation (Weller et al., 2007). Thus, conducting an initial heat acclimatization camp several weeks before the target event may increase the speed at which adaptations occurs in a follow-up pre-competition camp. For example, the main acclimatization block can be performed in the 2 weeks prior to travel, with 4–5 days of re-acclimation after arrival at the competition venue. Such planning accounts for the tapering need of the athlete before a major competition (Daanen et al., 2018). Thus, it may be more feasible if an acclimatization block was to be undertaken a few weeks before, followed by acute heat exposure throughout the taper to maintain the initially conferred adaptations. It is also possible to incorporate passive heat acclimation techniques such as sauna bathing (Scoon et al., 2007) and hot water immersion (Zurawlew et al., 2016) for 30–40 min pre- or post-training. This approach takes advantage of core temperature being elevated from training and can be further combined with extra (i.e., insulative) clothing during training to increase the stimulus. Immersion should be undertaken at water temperatures of around 40°C to induce adaptation while remaining tolerable (Figure 2). Although not as specific as exercise heat acclimatization *per se*, both passive and active methods of heat acclimation can be used to accommodate taper and travel requirements.

## Hydration for Performance and Recovery

Although, heat dissipation relies on sweat evaporation, a caveat to this may be the progressive dehydration that ensues, if sweat losses are not adequately replaced by fluid consumption (Sawka et al., 2007; Maughan and Shirreffs, 2010). While dehydration exacerbates heat stress (Sawka et al., 2015), the magnitude may be lower during real-world competition than previously estimated in a laboratory study (Goulet, 2013). Nevertheless, hydration prior to, during and following exercise is important for athletes to perform well and ensure their safety in the heat; particularly during the heat acclimatization period due to the increase in sweat rate. It should however be acknowledged that an acute increase in fluid absorption will result in an increase in urine excretion, and that the body will need a few days to adapt (Racinais et al., 2015a). During the acclimatization period, recovery drinks should include sodium to compensate for the sweat losses, whilst maintaining the usual requirements in carbohydrates and protein to optimize recovery (Racinais et al., 2015a). In this context, milk is a suitable recovery drink covering both the exercise recovery and re-hydration needs (Maughan et al., 2016).

During exercise, and especially competition, drinking to thirst has been shown to be adequate for exercise lasting between 1 and 2 h in cool environments (Kenefick, 2018). However, during exercise in the heat, a planned drinking strategy may further optimize performance, especially during high-intensity activities lasting longer than 90 min (Kenefick, 2018), for which athletes may require carbohydrates. “Heavy and salty” sweaters (e.g., with a sweat-rate of 3 L/h and a sweat sodium concentration of 70 mmol/L) may also need sodium supplementation (Racinais et al., 2015a). Drinking plans should be targeted toward preventing body mass losses exceeding 2–3%, but never to the extent where it might increase body weight (Kenefick, 2018), as over-hydration can result in serious (potentially deadly) hyponatremia (an imbalance of the salts in the body; Hew-Butler et al., 2015). Athlete should therefore measure their change in body weight during training in the heat simulating the competition to estimate their fluid need several weeks before competing. Hydration in distance running is however limited by the absorption limits of the gut (~1.2 L/h) and by the possibility to drink while running. The priority should therefore be on limiting pre-event dehydration and optimizing recovery.

## Warm-Up or Pre-Cooling Before Competition?

Warming-up before an intense exercise or a competition has numerous physiological and psychological benefits improving performance (Racinais et al., 2017). However, warming-up in a hot environment would exaggerate the increase in core temperature and limit heat-storage capacity, thereby affecting prolonged exercise performance in the heat (Racinais et al., 2017). Consequently, in order to minimize unnecessary heat exposure and heat gain, the warm-up should be tailored to the environmental conditions (e.g., warm-up in the shade, modified exercise types and intensity, lower duration) and combined with cooling aids.

**Figure 3:**
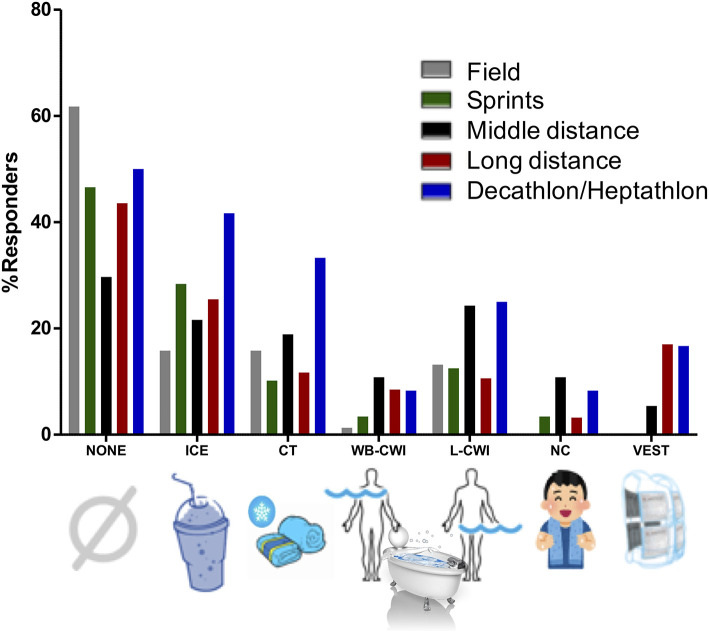
Intended use of cooling strategies at the 2015 Beijing IAAF World Athletics Championships (Périard et al., 2017). ICE; ice ingestion; CT, cold towels; WB-CWI, whole body cold water immersion; L-CWI, lower limb cold water immersion; NC, neck collar; VEST, cooling vest.

Many athletes commonly use pre-cooling techniques (Figure 3; Périard et al., 2017), a strategy that may seem counterproductive and potentially incompatible with warming-up. Pre-cooling refers to the lowering of pre-exercise body temperatures to lower thermal strain during the ensuing exercise task (Marino, 2002; Choo et al., 2018). A 0.5°C decrease in pre-exercise core temperature is often the desired goal of a pre-cooling strategy, whilst a 0.3°C decrease is generally considered the physiological minimum to confer a thermoregulatory advantage (Marino, 2002). Common pre-cooling modalities such as cold water immersion (CWI), ice slurry ingestion and the use of cooling vests have been shown to achieve such reductions in core temperature within 30–60 min (Marino, 2002; Ihsan et al., 2010; Ross et al., 2013; Zimmermann et al., 2018). Nevertheless, factors such as body mass and thermal responses (e.g., shivering) may influence the effectiveness of a particular pre-cooling strategy (Marino, 2002; Quod et al., 2006). It is therefore imperative that intended (race) pre-cooling strategies are incorporated and trialed during training, and selected based on athletes' comfort, effectiveness and available resources. The development of ingestible temperature monitoring capsules, as well as cooling aids (e.g., specialized slushy dispensers and bottles, portable water baths, lightweight ice vests) have by far eased the logistical constraints associated with on-field temperature monitoring and administration of pre-cooling strategies. As such, practitioners are encouraged to utilize such advances during training to formulate optimal pre-race cooling strategies. CWI is regarded as the most effective modality to reduce body temperatures (Ross et al., 2013; Choo et al., 2018). However, although advances in equipment/product design have improved the feasibility of this modality in the field, implementing it around competition venues will likely be cumbersome. Indeed, immersion temperatures need to be controlled between 20 and 25°C to minimize adverse thermal responses and discomfort (Marino, 2002; Quod et al., 2006), thus requiring manpower, as well as access to large volumes of water, ice, and/or electricity.

The use of cooling vests offers a more practical approach to undertake in the field. These garments are designed such that it covers the entire torso, and facilitates heat removal primarily through conduction. Albeit, the cooling capacity of such garments can be limited due to a contact interface of only 5–10% of the total body surface area (Luomala et al., 2012) failing to lower core temperatures when worn during normothermic rest (Duffield et al., 2003; Quod et al., 2008), they are still effective to limit excessive increases in core temperature during warm-up (Arngrïmsson et al., 2004; Hunter et al., 2006; Stannard et al., 2011; Katica et al., 2018). This is likely explained by the increase in circulation and heat production resulting from the warm-up, which may have better facilitated heat transfer to the vest. There may be some concerns though, regarding the potential increase in energy expenditure during warm-up, due to the added weight incurred by the vest. However, advances in technology and product design have resulted in vests weighing as little as 0.5 kg (Eijsvogels et al., 2014), with the commonly investigated vest by Artic Heat weighing 1.5 kg (Bogerd et al., 2010). Nevertheless, concerned athletes may opt to slightly reduce their warm-up running speed to compensate for the increased metabolic cost associated with utilizing the vest (Arngrïmsson et al., 2004), or combine with other practical strategies (described below), limiting the use of the vest during the more static components of the warm-up.

The ingestion of ice slurries or cold fluids is another practical modality which has gained considerable adherence amongst athletes (Figure 3; Périard et al., 2017). The underlying premise is that ingesting ice or cold fluids offer an additional avenue for heat transfer as the ingested bolus equilibrates with internal body temperature. The ingestion of an ice slurry over cold water has been suggested to increase the potential for heat transfer, owing to the additional thermal energy required to convert ice to liquid (334 kJ/kg). Approximately 7 g/kg body mass of ice has been shown to typically induce a core temperature decrease of about 0.5°C (Ihsan et al., 2010; Siegel et al., 2010; Zimmermann et al., 2018). It is a mobile and flexible strategy which can be used prior to, and/or assimilated into the warm-up routine. However, ice or cold fluid ingestion has been shown to markedly reduce sweat rates through afferent feedback from thermoreceptors within the gut or stomach regions, potentially decreasing the evaporative potential for heat loss (Morris et al., 2014, 2016). Thus, caution is warranted when considering the use of ice or cold fluids in competition environments with high evaporative potential (e.g., dry and windy environments); whereas, when evaporative heat loss is minimal (e.g., humid environments), the decrease in sweat rate by ice ingestion may be beneficial in preserving blood volume, whilst facilitating internal cooling. Overall, ice and cold fluid ingestion are appropriate strategies to be implemented during warm-up.

Pre-cooling interventions can be mixed [e.g., cooling vests (torso) plus cold towels (neck and head regions), while drinking ice slurry] as benefits are proportionate to the body surface area cooled and the duration of cooling (Duffield et al., 2009; Minett et al., 2011, 2012; Soo et al., 2019).

## Mitigating the Heat Stress During the Competition Itself

Cooling strategies may also be implemented during an event (i.e., mid-cooling) for immediate impact on performance (Stevens et al., 2017). While mid-cooling strategies may include the dousing of cold fluids on the head and facial regions, use of cooling garments or collar, as well as the ingestion of cold beverages or ice (Ansley et al., 2008; Minett et al., 2011; Stevens et al., 2013; Riera et al., 2014; Schulze et al., 2015; Sunderland et al., 2015; Périard et al., 2017); water dousing and ingestion are probably the only feasible strategies in athletics, and may be limited to endurance events on the road (i.e., marathon and race walks). Indeed, mid-cooling is not a relevant ergogenic aid for field, sprint and middle-distance events, and though may be relevant, not feasible in long distance track events. An exception might be the IAAF World Athletics Championships held in Beijing, where water stations were made available during the 5,000 m final. While the use of cold fluid ingestion and dousing can be seamlessly combined, the relative benefit of these strategies seem to be highly influenced by the environmental conditions. In humid environments, cold fluid ingestion, although reducing sweat response (Morris et al., 2014, 2016), may attenuate the increase in core temperature and minimize the decline in blood volume; whilst water dousing though offers a limited cooling effect (as evaporative potentials are highly diminished in humid environments) but may confer some perceptual benefits, although such advantages may be largely transient (Riera et al., 2014; Schulze et al., 2015; Tyler et al., 2015). Conversely, in dry and windy environments, water dousing increases the evaporative potential and hence body heat dissipation, whilst the ingestion of cold fluids has been shown to exaggerate the increase body temperatures by reducing sweat rates (Morris et al., 2014, 2016). Regardless, when deciding on ingestion and dousing strategies during race, it mainly depends on the immediate athlete needs: drink if thirsty, pour if hot (Morris and Jay, 2016).

Athletes should also protect their eyes by wearing UV ray blocking sun-glasses in a dark tint (i.e., grade 3) and their skin by using non-greasy sun-screen (water-based sun screen should be preferred to oil-based sun-screen that may affect sweating). Lightly colored clothing can also minimize the effect of the sun's radiation, but clothing should not impair sweat evaporation.

## Managing Exertional Heat Illness

Exertional heat stroke (EHS) is the most severe form of heat illness, typically characterized by a neuropsychiatric impairment coupled with a high core body temperature (>40.5°C). Athletes will likely have no sequela if their internal body temperature is reduced <40°C within 30 min (Stearns et al., 2017), but may however suffer permanent disability beyond this point and even death if treatment is postponed by more than 1 h.

In order to minimize the number of minutes the EHS patient is hyperthermic, it is crucial that EHS is considered as one of the primary possible diagnosis when an athlete has collapsed or is struggling during intense exercise in the heat. The other potential medical issues need to be quickly considered and excluded, including cardiac conditions, asthma, exertional hyponatremia, head injury, exertional sickling, diabetes, and anaphylaxis; and core body temperature should be assessed immediately to quickly confirm or invalid the EHS diagnosis. When athletes have been exercising intensely in the heat, it is essential that rectal temperature is utilized to determine if the athlete is severely hyperthermic or not (Casa et al., 2015). Signs of central Nervous System (CNS) dysfunction (e.g., confusion, altered consciousness, coma, convulsions, agitation, combativeness, disorientation) coupled with a rectal temperature >40.5°C (>105°F) indicate an EHS episode that needs immediate attention.

As soon as diagnosed, EHS should be immediately treated by CWI. Research has verified that CWI has the fastest cooling rates, hence should be the cooling mode of choice (Casa et al., 2015; Demartini et al., 2015). In controlled athletic venues such as practices, endurance sports events, and competitions conducted in warm/hot environments, it would therefore be recommended to have CWI set-up at convenient locations at the venue or course. A few tips to assure a successful immersion procedure include: consistently stir the water during cooling, cover as much skin surface area as possible, drape sheet under armpits to stabilize patient in tub, use rectal thermistor so that core temp can be monitored during cooling, and utilize water temperatures around 10–15°C (although a wide range of water temperatures will provide effective cooling rates). Remove from the CWI when the EHS patient reaches about 39°C. To minimize the duration at which an athlete remain >40.5°C, it is mandatory to cool-first/transport second (Belval et al., 2018). For instance, if an EHS patient needed to wait for the ambulance to be called/arrive/on-scene/transport/enter hospital/establish cooling at hospital; well over the established 30 min will be lost before even before aggressive cooling would begin. To increase the likelihood of a successful treatment outcome of EHS cases, it is essential to work with local ambulance/hospital services so that the concept of cool-first/transport second is firmly established as part of the medical policy prior to a time of crises.

## Conclusion

In summary, hot and humid ambient condition may play a major role in numerous athletic events. Fortunately, athletes have a toolbox of countermeasures with various efficacy and feasibility. The most important is to plan ahead by training in the heat for 2 weeks. Hydration should also be considered during the preparation and recovery phases as it is not always feasible to drink during the event. Depending on thirst and perception, the water available during competition may be ingested or doused around the head. Another important consideration is to limit unnecessary increases in core temperature by adapting the warm-up with some cooling strategies such as ice-vest or cold/iced drinks, and avoiding clothing or accessories limiting sweat evaporation. Medical response should focus on rapid cooling by having CWI tubs already set-up to cool first / transport second.

## Author Contributions

All authors listed have made a substantial, direct and intellectual contribution to the work, and approved it for publication.

### Conflict of Interest

SR and MI are part of Aspetar, the medical provider of the upcoming world championship. SR also collaborates with the IAAF on several projects including a leaflet distributed to the athletes containing a part of the information presented here. The remaining authors declare that the research was conducted in the absence of any commercial or financial relationships that could be construed as a potential conflict of interest.
